# Vial usage, device dead space, vaccine wastage, and dose accuracy of intradermal delivery devices for inactivated poliovirus vaccine (IPV)

**DOI:** 10.1016/j.vaccine.2016.11.098

**Published:** 2017-03-27

**Authors:** Courtney Jarrahian, Annie Rein-Weston, Gene Saxon, Ben Creelman, Greg Kachmarik, Abhijeet Anand, Darin Zehrung

**Affiliations:** a PATH, PO Box 900922, Seattle, WA 98109, USA; b Centers for Disease Control and Prevention, 1600 Clifton Rd, Atlanta, GA 30333, USA

**Keywords:** Inactivated polio vaccine, Intradermal delivery, Vaccines, Delivery devices, Vaccine wastage, AD, autodisable, DSJIs, disposable-syringe jet injectors, fIPV, fractional inactivated poliovirus vaccine, ID, intradermal, IM, intramuscular, IPV, inactivated poliovirus vaccine, OPV, oral poliovirus vaccine, tOPV, trivalent OPV, bOPV, bivalent OPV, UNICEF, United Nations Children’s Fund, WHO, World Health Organization

## Abstract

**Introduction:**

Intradermal delivery of a fractional dose of inactivated poliovirus vaccine (IPV) offers potential benefits compared to intramuscular (IM) delivery, including possible cost reductions and easing of IPV supply shortages. Objectives of this study were to assess intradermal delivery devices for dead space, wastage generated by the filling process, dose accuracy, and total number of doses that can be delivered per vial.

**Methods:**

Devices tested included syringes with staked (fixed) needles (autodisable syringes and syringes used with intradermal adapters), a luer-slip needle and syringe, a mini-needle syringe, a hollow microneedle device, and disposable-syringe jet injectors with their associated filling adapters. Each device was used to withdraw 0.1-mL fractional doses from single-dose IM glass vials which were then ejected into a beaker. Both vial and device were weighed before and after filling and again after expulsion of liquid to record change in volume at each stage of the process. Data were used to calculate the number of doses that could potentially be obtained from multidose vials.

**Results:**

Results show wide variability in dead space, dose accuracy, overall wastage, and total number of doses that can be obtained per vial among intradermal delivery devices. Syringes with staked needles had relatively low dead space and low overall wastage, and could achieve a greater number of doses per vial compared to syringes with a detachable luer-slip needle. Of the disposable-syringe jet injectors tested, one was comparable to syringes with staked needles.

**Discussion:**

If intradermal delivery of IPV is introduced, selection of an intradermal delivery device can have a substantial impact on vaccine wasted during administration, and thus on the required quantity of vaccine that needs to be purchased. An ideal intradermal delivery device should be not only safe, reliable, accurate, and acceptable to users and vaccine recipients, but should also have low dead space, high dose accuracy, and low overall wastage to maximize the potential number of doses that can be withdrawn and delivered.

## 1 Introduction

The oral poliovirus vaccine (OPV), a live attenuated vaccine, has been the principal tool for polio eradication since the start of the Global Polio Eradication Initiative. However, polioviruses in OPV are live and attenuated and in very rare cases can acquire neurovirulence, causing paralysis similar to wild polioviruses. Eradication of all poliovirus strains will require the eventual cessation of OPV use. The World Health Organization (WHO) Strategic Advisory Group of Experts on Immunization recommended a phased cessation of OPV, starting with type 2 virus cessation by replacing trivalent OPV (tOPV) with bivalent OPV (bOPV), with the simultaneous introduction of trivalent inactivated poliovirus vaccine (IPV) in routine immunization [1].

There are currently four manufacturers globally of Salk IPV, of which two are major suppliers (Sanofi Pasteur and Bilthoven/Serum Institute of India) to the United Nations Children’s Fund (UNICEF). Additional manufacturers have Sabin IPV vaccines in development, and one of these has licensure in China. In preparation for the switch to bOPV in April 2016, countries that were not using IPV in their routine immunization schedule were expected to introduce at least one dose of IPV in advance [2], [3]. This has increased IPV demand from about 80 million doses in 2013 to about 200 million doses in 2016 [4]. In addition, IPV campaigns are being conducted to interrupt wild poliovirus transmission. Together, the rapid increase in demand of IPV for routine immunization in concert with demand of IPV for campaigns in poliovirus endemic countries and temporary reductions in expected manufacturing capacity has stretched IPV supply and has delayed introduction of IPV in advance of the switch from tOPV to bOPV [5], [6].

IPV is substantially more expensive than OPV (UNICEF procurement price of US$1 to $2 per IPV dose vs $0.15 per OPV dose) [5], [7], and the polio eradication program is exploring ways to make IPV affordable, especially for low-resource countries [8]. One of the options is to reduce the antigen content of each dose by administering a fractional intradermal (ID) dose of 0.1 mL of IPV, instead of the standard dose of 0.5 mL delivered IM. A number of clinical studies have compared ID delivery of reduced doses of IPV to full-dose IM delivery with variable results depending on the vaccination regimen used [9], [10], [11], [12], [13], [14], [15], [16], [17], [19], [20], [21].

Intradermal IPV, with only one-fifth of the full IM dose of antigen required, also offers the ability to stretch the limited supplies of IPV. WHO’s position paper on polio vaccines recommends that countries consider introduction of intradermal fractional IPV (fIPV) with two doses at 6 and 14 weeks of age [22], [23], as two doses of fIPV have been shown to be more immunogenic than one IM dose of 0.5 mL IPV [16], [19]. With this new recommendation, some countries have started introduction of fIPV in routine childhood immunization, including several states in India which introduced fIPV in April 2016 [24] as well as Sri Lanka [25]. The Global Polio Eradication Initiative has also stated that IPV campaigns, such as those in response to type 2 vaccine derived polio virus (VDPV2) outbreak after the switch from tOPV to bOPV, must utilize fIPV [22], [26], which has been implemented in a campaign in June 2016 in Hyderabad, India [27]. ID delivery could also have ancillary benefits, including easing the logistical burden of immunization programs by reducing the amount of cold chain space required for vaccine storage and transport [28]. In addition, several ID devices in development are needle-free and could eliminate the risk of needle-stick injuries as well as the costs associated with sharps waste disposal [29], [30], [31].

The principal reason for use of ID fIPV in the polio program is the ability of fIPV to stretch existing supplies of IPV and offer improved type 2 immunogenicity. Therefore, careful selection of an ID delivery device that is suitable for the intended scenario of use, minimizes wastage, and enables consistent delivery of an accurate dose will be essential. A vaccine vial containing a single 0.5-mL IM dose of IPV may become a five-dose vial for 0.1-mL ID injections, and vials containing five or ten IM doses could theoretically provide 25 or 50 ID doses. However, depending on the design of the ID device used, a vial could supply fewer or greater than the nominal number of doses; multiplied out over the course of several million vaccinations during a large campaign, these small differences can add up significantly. Several factors make a difference in the true number of doses available from a multiple-dose vial: device dead space (fluid retained in the syringe after injection), filling technique, use of a vial adapter versus a filling needle, overfill volume, and accuracy of dose delivered. The purpose of this study was to characterize the relative differences in wastage and dead space between a range of commercially available and prototype ID delivery devices that may potentially be used for delivery of IPV and how those differences would affect the number of 0.1-mL ID doses that can be drawn from a single-dose IM IPV vial.

A variety of existing and novel ID delivery technologies have been investigated for delivery of IPV [32], [33]. These can be grouped into two categories: devices which can deliver the current, liquid formulation of IPV (conventional needles and syringes, ID adapters, mini-needles, hollow microneedles, and disposable-syringe jet injectors [DSJIs]) and delivery technologies that incorporate the vaccine in a solid form, such as microarray patches. This report focuses on devices for delivery of the liquid vaccine, which offer a shorter-term solution to the issue of IPV cost and supply, and do not require reformulation of existing vaccines. Microarray patch technologies in development for IPV may potentially offer additional benefits (thermostability, ease of delivery, elimination of sharps waste) and have a longer-term timeline to availability. Since microarray patches do not deliver the current liquid vaccine, they were out of scope for this investigation [34], [35].

**Fig. 1:**
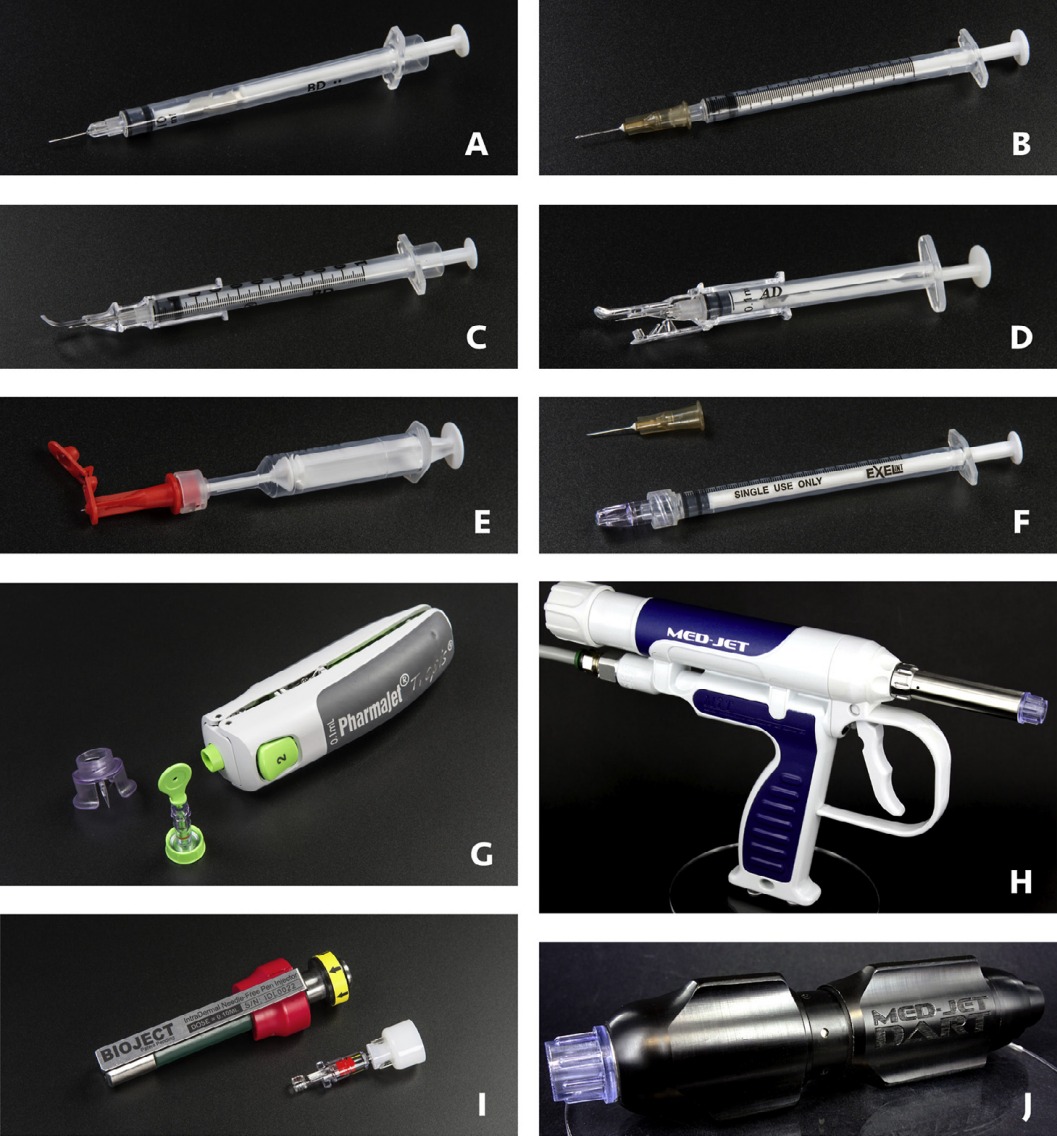
ID delivery devices that can be filled on site and used to deliver liquid IPV. (A) BD 0.1-mL SoloShot Mini autodisable syringe; (B) BD 1-mL luer-slip syringe; (C) BD 1-mL allergy syringe with West ID adapter; (D) Helm 0.1-mL autodisable syringe with prototype autodisable ID adapter; (E) Star ID syringe with mini-needle; (F) NanoPass MJ600 hollow microneedle, EXEL 1-mL luer-lock syringe, and filling needle; (G) PharmaJet Tropis with filling adapter and needle-free syringe; (H) MIT Canada Med-Jet® H4; (I) Bioject ID Pen with needle-free syringe; (J) MIT Canada Med-Jet® Dart.

The Mantoux method is the accepted technique used for ID delivery with a needle and syringe and is currently used worldwide to deliver bacille Calmette-Guérin and rabies vaccines, as well as some pharmaceuticals, particularly those used for tuberculin and allergy testing. Syringes used in different scenarios for ID delivery include fixed-dose autodisable (AD) syringes, allergy and insulin syringes with staked needles, and syringes with detachable luer-slip needles, and each of these is broadly available and inexpensive. However, the Mantoux method requires training to acquire the skill to consistently and reliably accomplish the ID injection, as a health care worker must insert the needle at a 5- to 15-degree angle and inject the fluid just below the skin’s surface to create a wheal indicating a successful injection [36]. To overcome the perceived difficulty of using the Mantoux method, a variety of ID delivery devices have been developed to improve both the ease of use and accuracy of ID delivery (Fig. 1). These include an ID adapter (West Pharmaceutical Services, Inc.) that fits over a standard syringe with a staked needle and limits the depth and angle of the needle during injection [37], [38]. Other alternatives are a syringe with a mini-needle 1.5 mm in length which enables perpendicular injections (Star Syringe) [39], and also devices consisting of single or multiple hollow microneedles (less than 1 mm in length) affixed to a luer-slip hub for mounting on the syringe, such as the NanoPass MicronJet600 (MJ600) [40] and the DebioJect microneedle device [41]. In addition, prefilled ID devices with mini-needles have been developed, such as the BD Soluvia® injection system (approved for delivery of inactivated influenza vaccine [42]) and the Novosanis VAX-ID [43], although due to increased costs and cold chain volume of prefilled technologies, they are likely to be less appropriate for IPV delivery in low- and middle-income countries [29].

DSJIs use gas or spring power to generate a high-pressure stream of liquid through an orifice in a needle-free syringe to penetrate the skin. Jet injectors for subcutaneous and IM delivery have regulatory clearance for delivery of vaccines, and one device, the PharmaJet Stratis, has been WHO prequalified [44]. DSJIs that are specific for ID delivery and designed with features suitable for use in low-resource settings have also been developed, including the PharmaJet Tropis, Bioject ID Pen, and MIT Canada Med-Jet Dart and H4 (Fig. 1) [9].

## 2 Materials and methods

**Fig. 2:**
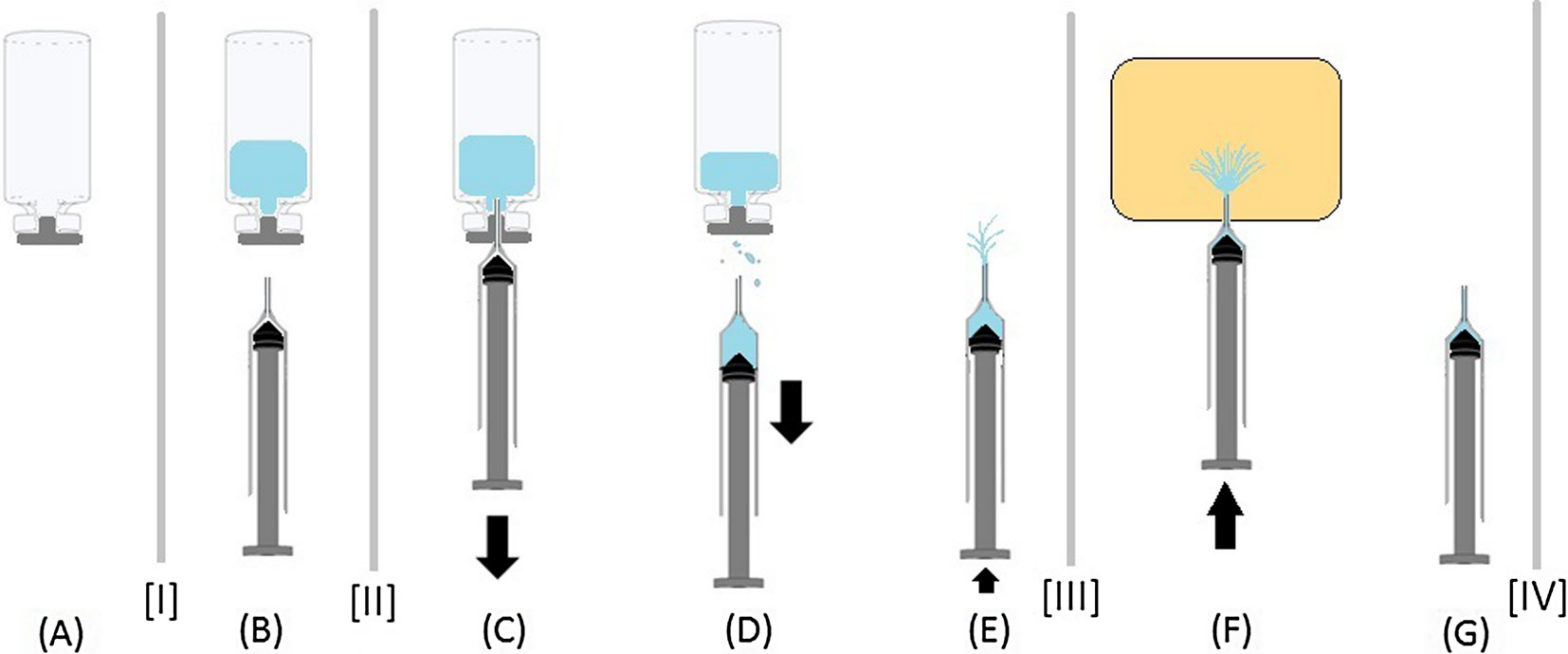
Schematic of testing process. (A) Empty vial; (B) Filled vial and empty delivery device; (C) Withdrawing deionized water from a filled vial; (D) Withdrawing device from vial (note potential wastage on withdrawal); (E) Setting of dose to 0.1 mL into the air (note wastage); (F) Delivery of dose; (G) Wastage due to filled dead space in syringe.

ID delivery devices were tested in PATH’s product development workshop in accordance with methods described in ISO 7886-1:1993 (Sterile Hypodermic Syringes for Single Use, Annex C, Method for Determination of Dead Space). Volumes were calculated by means of weight measurements converted using the density of distilled water at room temperature (0.998 g/mL). Using a calibrated digital scale (Mettler Toledo XS304) accurate to ±0.1 mg, separate weight measurements were taken of both the vial (with capping materials) and delivery device at points seen represented by the dividing lines between steps (Fig. 2, marked [I], [II], [III], and [IV]). Weight differences between measurement points during the delivery process indicate key characteristics such as:

Deadspace=deviceweightat[IV]-deviceweightat[I]

Retainedvialamount=vialweightafterlastwithdraw[III]-emptyvialweight

Delivereddosevolume=deviceweightat[III]-deviceweightat[IV]

Glass vials (13 mm × 31 mm, 2-mL volume, Schott Pharmaceutical) for each test device were filled with 0.700 mL of deionized water (the volume of a standard single-dose IM IPV vial, including 0.2 mL overfill) using a calibrated micropipette accurate to ±1 μL and sealed with a 13-mm septum (Kimble Chase) and cap (Wheaton). Doses were withdrawn and delivered according to the instructions for use (into a beaker) until no more water could be drawn from the vial. This entire process was repeated with five vials for each test condition.

ID devices that have been previously evaluated for delivery of IPV and that are designed to be filled on site with liquid vaccine were selected for testing (approximately 25 units evaluated per device, depending on how many were required to draw all doses in five vials). These included an AD syringe with staked needle (BD 0.1-mL SoloShot Mini AD syringe with 27-gauge 3/8″ needle [Fig. 1A]); a selected syringe with luer connection and separate needle (BD 1-mL luer-slip syringe with BD 27-gauge 1/2″ needle [Fig.1B]); two syringes with staked needles that are compatible with versions of the ID adapter (BD 1-mL allergy syringe with 27-gauge 1/2″ needle [Fig.1C] and Helm 0.1-mL AD syringe with 27-gauge 1/2″ needle [Fig.1D]); a mini-needle syringe (Star ID syringe, Fig.1E); a hollow microneedle hub attached to a luer syringe (NanoPass MJ600 and EXEL 1-mL luer-lock syringe [Fig.1F]); and several DSJIs (PharmaJet Tropis [Fig.1G], MIT Canada Med-Jet H4 [Fig.1H], Bioject ID Pen [Fig.1I], and MIT Canada Med-Jet Dart [Fig.1J]). The MIT Canada Dart and Med-Jet H4 use the same disposable needle-free syringe and therefore the results for this needle-free syringe apply to both of these DSJI devices. The needle and syringes, ID adapter, and MJ600 devices have regulatory clearances, while the jet injectors tested and the Star ID syringe were investigational prototypes in development.

The conventional syringes were filled with their own needles. Jet injector needle-free syringes were filled using filling adapters provided by the device manufacturers in accordance with the device instructions for use. The Star ID syringe was filled using the plastic spike integrated into the syringe body. The EXEL luer-lock syringe for the NanoPass MJ600 device was filled using a separate BD 27-gauge 1/2″ length needle, which was then removed before attachment of the MJ600 hollow microneedle hub.

Vaccine wastage during filling and injection can occur at multiple points during the delivery process:

• Device dead space: The internal geometry of the delivery device can include dead space that retains some vaccine after the dose is delivered.
• Filling process and user technique: Wastage can occur during the filling process, such as drops remaining on the exterior of the components or in the vial adapter or filling needle. User/device instructions can also vary in the technique used to set the correct dose and remove air bubbles (either by injecting the vaccine back into the vial or out into the air).

Two filling procedures for setting the dose and removing bubbles were evaluated to determine the potential effect of user filling technique on overall wastage. In the first technique, the needle was kept in the vial throughout the process and the overfill and bubbles in the syringe were returned to the vial. In the second technique, after withdrawing the dose from the vial, the overfill and bubbles in the syringe were ejected into the air (Fig.2E). Training materials on safe injection techniques for vaccinators recommend both techniques [45], [46]. The delivery devices were primarily tested using only the more conservative “return to vial” filling technique, with the exception of the Star ID syringe, where the dose was set using the “eject in air” technique in order to remain consistent with the manufacturer’s instructions. The BD SoloShot Mini and BD 1-mL luer-slip syringe were tested using both techniques to evaluate the potential variability in user technique. Results presented in this paper used the “return to vial” filling technique, except for the Star ID syringe, unless otherwise noted.

The average number of ID doses per single IM dose vial (fill of 0.7 mL) was calculated directly from the number of full doses withdrawn. WHO recommends that partial doses remaining in a vial should be discarded and should not be combined with vaccine from a new vial for injection safety reasons [47]. Therefore, in this study the partial doses remaining in each vial were measured but not included in the number of doses obtained from a vial.

From the measured weights, the volume delivered (V_Delivered_), total wastage (V_Wastage_), volume remaining in vial (remainder of vial content that cannot be removed) (V_Remaining in vial_), and delivery device dead space were calculated. These values were then used to estimate the number of ID doses per vial for different vial sizes (5-dose vial with 3.0 mL fill and 10-dose vial with 5.5 mL fill) according to the following equation:

$$\mathrm{Estimated}\;\mathrm{ID}\;\mathrm{doses}\;\mathrm{per}\;\mathrm{vial}=\frac{V_{\mathrm{Total}}-V_{\mathrm{Remaining}\;\mathrm{in}\;\mathrm{vial}}}{\left(V_{\mathrm{Delivered}}+V_{\mathrm{Wastage}}\right)}$$

The potential percentage difference in vaccine purchase quantity (and cost) to cover a given population of children using each ID delivery device was estimated in comparison to the nominal number of ID doses that one might expect to obtain based on fractional dosing (5 ID doses from an IM single-dose vial; 25 ID doses from an IM five-dose vial; 50 ID doses from an IM ten-dose vial). This percentage was calculated by dividing the nominal number as defined above by the estimated doses per vial for each device. All calculations were done in Microsoft Excel.

## 3 Results

### 3.1 Delivered dose volume

**Fig. 3:**
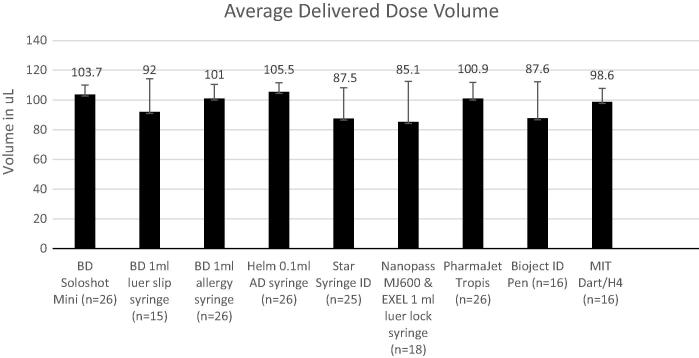
Mean delivered dose volume per device. Error bars represent one standard deviation.

The delivered dose volume for all devices ranged from a mean of 85.1 μL to 105.5 μL for the devices tested (Fig. 3). The precision of the dose volume also varied, with standard deviations ranging from 6.1 μL to 27.4 μL, which reflects the large degree of variability between both devices and technique prescribed by the instructions for use.

### 3.2 Device dead space and filling procedure wastage

**Fig. 4:**
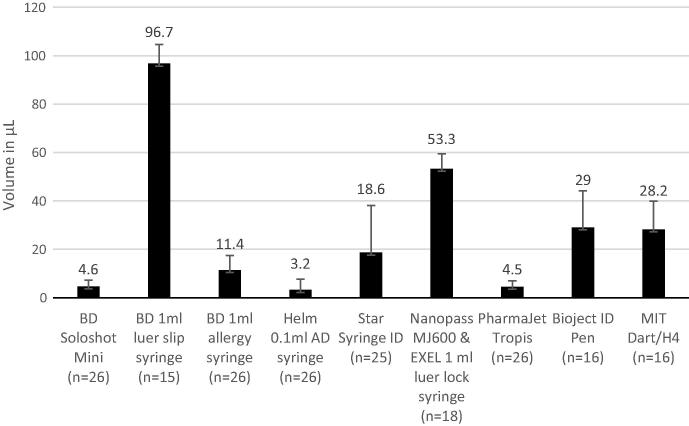
Mean dead space per device. Error bars reflect standard deviation.

The mean dead space of the devices tested ranged from 3.2 μL to 96.7 μL per injection (Fig. 4). The vial-retained volume fluctuated widely between individual vials and ranged from 17 μL to 312 μL per vial, but no significant differences between devices were observed (data not shown).

Use of a vial adapter to fill the DSJI devices added a mean 14 μL of vaccine wastage per vial adapter used, and using a separate filling needle for the Nanopass MJ600 device added 16 μL. Using the “eject in air” technique for removing air bubbles from the syringe resulted in more vaccine wastage per injection as compared to the “return to vial” technique. The mean increase in vaccine wastage for the “eject in air” technique was 13.7 μL for the BD SoloShot Mini and 23.4 μL for the BD luer-slip syringe.

### 3.3 Doses obtainable per vial

The mean number of complete doses obtained from a vial ranged from 3.0 to 5.2 (see Table 1). Averages greater than the nominal amount of fractional ID doses that should be available (five 0.1-mL doses from a single-dose IM vial) reflected retrieval of 6 complete doses in some tests, due to the overfill that vaccine manufacturers incorporate. Extrapolations from this data suggest that the number of ID doses available from a five-dose IM vial could range from 15.8 to 27.1, and that 29.8 to 50.8 ID doses might be obtainable from a ten-dose IM vial. Compared to the nominal number of fractional doses that could be anticipated (5 ID doses from an IM single-dose vial; 25 ID doses from an IM five-dose vial; 50 ID doses from an IM ten-dose vial), additional quantities of vaccine would need to be purchased to cover the same population if devices with high wastage rates were used. In some cases modeled, this had the effect of doubling vaccine purchase costs (Table 1).

**Table 1:** Number of full intradermal doses that could be obtained from an IM single-dose vial (average from test data), and number of full doses per IM five-dose and ten-dose vials (theoretically calculated). Relative vaccine purchase costs were compared to the nominal number of ID doses that could be expected based on fractional dosing (5 ID doses from an IM single-dose vial; 25 ID doses from an IM five-dose vial; 50 ID doses from an IM ten-dose vial) and exclude the costs of the delivery device.

Category	Delivery device	Single-dose IM vial		Five-dose IM vial		Ten-dose IM vial	
		Number of ID doses	Relative vaccine purchase cost	Number of ID doses	Relative vaccine purchase cost	Number of ID doses	Relative vaccine purchase cost
Needle and syringe	BD SoloShot Mini	5.2	96%	26.4	95%	49.4	103%
	BD 1-mL luer-slip syringe	3.0	140%	15.8	137%	29.8	201%

ID adapter	BD 1-mL allergy syringe	5.2	95%	27.1	92%	50.8	96%
	Helm 0.1-mL AD syringe	5.2	91%	26.7	93%	49.9	100%

Mini-needle	Star Syringe ID	5.0	97%	25.0	100%	46.7	117%

Hollow microneedle	NanoPass MJ600 & EXEL 1-mL luer-lock syringe	3.6	127%	17.9	128%	33.4	183%

DSJI	PharmaJet Tropis	5.0	99%	25.4	98%	47.6	112%
	Bioject ID Pen	3.8	124%	19.1	124%	35.7	172%
	MIT Canada Dart/H4	4.1	118%	19.6	122%	36.4	168%

## 4 Discussion

Due to the nature of the filling procedures and the design of the devices, wastage varied greatly from device to device. Device dead space was the greatest source of variation between devices, and can be attributed to the internal geometry of each device or the syringe used with it. In addition, for each type of device, there was fluid left in the vial that could not be withdrawn (vial- retained volume). Jet injector devices, such as the PharmaJet Tropis and the MIT Canada Dart and Med-Jet H4, and syringe-mounted, hollow microneedle devices, such as the NanoPass MicronJet600, use a vial adapter or filling needle to draw doses from the vial. Other contributors to overall wastage were fluid retained in the dead space of the vial adapter or filling needle or lost during the filling process.

Dose accuracy also affected the number of doses that could be drawn from a vial, and, more importantly, could also impact the immune response to vaccination. Based on our data, there is a risk of under-dosing of 20 percent or more when a vaccinator has to measure a dose of 0.1 mL on a syringe labeled for a range of doses up to 1 mL. Fixed-dose devices that automatically set the dose volume, such as the PharmaJet Tropis and the BD SoloShot Mini and Helm AD syringes, tended to be more accurate and more precise in dosing than variable-dose devices that rely on the user to set the dose. It should be noted that this study measured dose accuracy only as the quantity ejected from the delivery device, which may not be the same as the quantity retained in the vaccine recipient, due to the potential for leakage out of the injection site or delivery of part of the dose to the exterior of the skin, which can vary between ID devices and users and may have an impact on immunogenicity [37], [38], [48], [49].

A key dose-sparing statistic is the number of doses that can be successfully drawn from each vial. From the data collected during this trial, the staked-needle syringes (BD SoloShot, BD allergy syringe, and Helm AD syringe, which can be used on their own for Mantoux injections, and the latter two also used with the West ID adapter), the Star ID syringe, and the PharmaJet Tropis syringe drew the largest number of complete doses, averaging at least five doses per vial. The devices that drew the least number of doses included the NanoPass MicronJet600 and the BD 1-mL luer-slip syringe, which obtained 3.6 and 3.0 doses per vial, respectively. The major difference between these devices and those that drew more doses was the amount of dead space present in the luer hub and needle interface. Luer syringes tend to have larger dead space volumes than staked-needle syringes due to the volume of fluid retained in the hub portion of the syringe. The EXEL 1-mL luer-lock syringe used with the NanoPass MicronJet600 device is designed to have a relatively lower dead space volume compared to the BD 1-mL luer-slip syringe and other commercially available luer syringes but still resulted in more vaccine wastage than non-luer devices. Unlike the PharmaJet Tropis syringe, which has a flat front interface with its filling adapter, the Bioject ID Pen and MIT Canada Dart syringes have luer-like nozzles that interface with a luer-fit filling adapter, which likely contributed to their larger dead space and fewer doses drawn from the vial.

Limitations of this study included the relatively small scale (approximately 25 devices per test condition), but this study size was chosen to be sufficient to identify major differences between devices. Single-dose vials were used for testing, but because five- and ten-dose IPV vials are predominantly available to low- and middle-income countries, calculations were used to estimate the potential impact of wastage on different vial sizes. The laboratory setting does not replicate conditions found in field use. Water was used instead of actual vaccine, but it has a similar viscosity as IPV, and so would be expected to perform similarly. The experimenters in this study attempted to put a reasonable effort into withdrawing doses toward the end of a vial’s capacity so as to recreate a clinical setting as closely as possible. Though this study was not designed to investigate person-to-person variability, user technique during filling will clearly play a critical role in the number of doses that can be drawn from a standard vial, based on the differences observed in the “eject in air” and “return to vial” techniques for removal of bubbles. Larger scale studies involving health care workers in a clinical setting will be important to validate the results and assess variability between users. Other than the commercially available needles and syringes, the ID adapter and the MicronJet600, the ID delivery devices tested were prototypes in development. Modifications to the designs and use methods of the devices tested could help improve dose accuracy and reduce wastage. Potential changes include reducing dead space inside the syringe luer, the vial adapter or filling needle; developing a fixed-dose rather than variable-dose design; and modifying the interaction of the device with the vial to reduce loss during the filling procedure and access more of the contents of the vial.

Although each individual injection may result in wastage of a very small amount of fluid, the importance of such wastage becomes evident when a large-scale introduction of ID delivery of IPV is considered. The number of doses obtainable from a multidose vial varies between ID devices by up to 42 percent, which could have a correspondingly large impact on the number of doses that must be supplied and, therefore, is a critical factor in the total cost of vaccination and the degree of supply stretching that is possible with ID delivery [32]. It will be important to pay careful attention to dead space and vaccine wastage in the design of ID delivery devices and the selection of devices and techniques for immunization program use, for IPV as well as for other vaccines that are currently delivered ID (such as rabies vaccine) and for others which may be considered for ID delivery in the future.

## 5 Conclusions

Our results suggest that there are large differences in dead space, overall wastage, dose accuracy and total number of doses that can be achieved depending on the device used. In general, we found that syringes with a staked needle could achieve a greater number of doses than syringes that require a luer lock or luer slip. Intradermal jet injectors could also achieve a total number of doses comparable to staked needle and syringe devices, depending on their design. Modifications of some ID devices could increase their efficiency of vaccine usage.

The decision whether to utilize ID delivery of IPV in an outbreak response or campaign setting or in routine immunization, and which ID delivery device to use, will be made by the WHO, regulatory authorities, and national immunization programs. Key factors in choosing an ID delivery device for IPV include cost, ease of use, vaccine and delivery device regulatory clearance, and clinical safety and immunogenicity data, which are available for IPV delivery with some devices tested in this analysis, such as the NanoPass MJ600 [14], [19] and PharmaJet Tropis [9] and are in progress for the ID adapter and Star ID Syringe. However, device wastage and dose accuracy information can also help inform selection of an intradermal delivery device for IPV or any other ID delivered vaccine. It will be important for immunization programs to select an intradermal delivery device that has low dead space and low overall wastage in order to maximize the potential number of doses that can be drawn and delivered.

## Conflict of interest

None reported.
